# A Smartphone App for Patients With Acute Coronary Syndrome (MoTER-ACS): User-Centered Design Approach

**DOI:** 10.2196/17542

**Published:** 2020-12-18

**Authors:** Nazli Bashi, Marlien Varnfield, Mohanraj Karunanithi

**Affiliations:** 1 Australian eHealth Research Centre Commonwealth Scientific and Industrial Research Organisation Brisbane Australia; 2 School of Medicine The University of Queensland Brisbane Australia

**Keywords:** mobile health, mHealth, mobile health apps, smartphone, mobile phone, self-management, patient education, cardiovascular disease, acute coronary syndrome

## Abstract

**Background:**

Postdischarge interventions are limited for patients with acute coronary syndrome (ACS) due to few scheduled visits to outpatient clinics and the need to travel from remote areas. Smartphones have become viable lifestyle technology to deliver home-based educational and health interventions.

**Objective:**

The aim of this study was to develop a smartphone-based intervention for providing postdischarge support to patients with ACS.

**Methods:**

The content of Mobile Technology–Enabled Rehabilitation for Patients with ACS (MoTER-ACS) was derived from a series of small studies, termed prestudy surveys, conducted in 2017. The prestudy surveys were conducted in Prince Charles Hospital, Queensland, Australia, and consisted of questionnaires among a convenience sample of patients with ACS (n=30), a focus group discussion with health care professionals (n=10), and an online survey among cardiologists (n=15). Responses from the patient survey identified educational topics of MoTER-ACS. The focus group with health care professionals assisted with identifying educational materials, health monitoring, and self-management interventions. Based on the results of the cardiologists’ survey, monitoring of symptoms related to heart failure exacerbation was considered as a weekly diary.

**Results:**

The MoTER-ACS app covers multimedia educational materials to adopt a healthy lifestyle and includes user-friendly tools to monitor physiological and health parameters such as blood pressure, weight, and pain, assisting patients in self-managing their condition. A web portal that is linked to the data from the smartphone app is available to clinicians to regularly access patients’ data and provide support.

**Conclusions:**

The MoTER-ACS platform extends the capabilities of previous mobile health platforms by providing a home-based educational and self-management intervention for patients with ACS following discharge from the hospital. The MoTER-ACS intervention narrows the gap between existing hospital-based programs and home-based interventions by complementing the postdischarge program for patients with ACS.

## Introduction

Acute coronary syndrome (ACS) includes a broad spectrum of clinical presentations of ST-segment elevation and non–ST-segment elevation myocardial infarction (MI) and different types of angina. Coronary heart disease affects 7.7% of Australians, and it is the leading cause of total burden of disease across life stages and consequently one of the most common causes of medical admissions [1]. In 2015, 61,600 people had an acute coronary event in the form of a heart attack or unstable angina, which equates to around 170 events every day. Furthermore, coronary heart disease contributed to 12% of all deaths in Australia in 2016 [2]. This high number of hospital admissions could be reduced by providing education, health monitoring, and support. However, due to providers’ workloads, the time-intensive nature of health services that provide face-to-face appointments, and the high number of remote and outer metropolitan patients, many Australian health services can only provide follow-up by phone for the majority of patients with ACS [3].

Currently, the ACS postdischarge model of care places limitations on conducting a full assessment and efficient follow-up of a patient’s condition and progress. Traditional postdischarge models include fixed appointments for clinical review regardless of patients’ requirements [4,5]. These episodic reviews do not provide clinicians with sufficient and updated information about a patient’s condition, as they may not happen at the right time to address the patient’s requirements. Furthermore, traditional hospital-based health care programs require significant resources, with patients experiencing long waiting times; these resources could be more appropriately used if prioritized based on patients’ needs [6]. A new ambulatory care model is required to enable discharged patients to receive either traditional face-to-face follow-up, especially if they have high risks and needs, or a home-based approach [5].

Advances in mobile communication technologies and medical devices enable mobile health (mHealth) interventions to combine the accessibility of home-based rehabilitation with the clinical expertise, supervision, and coaching that has traditionally been limited to hospital-based practices [7]. Previous research demonstrated a positive impact of remote patient monitoring, including mHealth, on patients with cardiovascular disease, mainly heart failure [8-10]. However, little is known about the feasibility and effects of smart phone–based remote monitoring on the outcomes of patients with ACS. If the mHealth intervention encourages homebound patients to actively engage in their disease management by self-monitoring physiological parameters under the supervision of health care professionals and adhering to medications, then the mHealth intervention has potential to improve patient health outcomes and quality of life [11,12]. The development of such interventions requires an iterative process of obtaining information and guidance from all stakeholders, including patients, software engineers, and health care providers [13]. The aim of this study was to develop a smartphone-based intervention to provide postdischarge support to patients with ACS. In this study, we involved stakeholders in prestudy surveys to develop a theory-based intervention that provides health monitoring, education, and support. This paper first defines the design objectives and theoretical framework of an mHealth postdischarge intervention, then describes the steps we performed to extend a mobile technology–enabled rehabilitation platform and its key components to postdischarge support of patients with ACS.

## Methods

### Design Objectives

Mobile Technology–Enabled Rehabilitation (MoTER) is a platform designed for home-based monitoring in cardiac rehabilitation using a smartphone app and the web [14]. The platform consists of a smartphone app (Android or iOS) with educational videos, health measures, exercise review, goal setting, motivational text messages, and a web portal. We extended the platform to create MoTER for patients with ACS (MoTER-ACS), which integrates a smartphone app (Android or iOS), Bluetooth-enabled devices (blood pressure [BP] cuff and body weight [BW] scale), and a web portal to provide health monitoring, evidenced-based education, and health care provider support for patients with ACS following discharge from the hospital. Furthermore, we aimed to optimize the app’s usability and to simplify navigation by including different levels of menu choices, creating a highly engaging appearance using quality images and adequate text, and reducing app errors through frequent testing. To achieve these aims, we studied mHealth apps by conducting literature reviews, including a systematic review on mHealth strategies and structures [15].

### Core Components of Secondary Prevention

Based on the Australian guidelines [16], interventions to reduce modifiable risk factors for cardiovascular disease (CVD) include advice and treatment on (1) smoking cessation, (2) maintenance of normal BP, (3) lowering of the serum cholesterol concentration and maintenance of the lipid profile within guideline levels, (4) lowering of serum glucose to within guideline levels, and (5) information on lifestyle risk factors, such as physical activity, diet, obesity, and alcohol consumption. The core components of secondary prevention include health evaluation, patient education, exercise training, lifestyle risk factor management, medication, and psychosocial management [16]. In this study, we aimed to address these components in the development of an mHealth intervention for postdischarge management of patients with ACS.

### Self-Efficacy Theory

Theory-based interventions have been demonstrated to be more effective than those without theoretical underpinnings. Applying behavior change theories to mHealth intervention designs significantly increases the likelihood of success [9]. The use of a theoretical framework enables researchers to address the complexity of mHealth interventions when involving a diverse set of stakeholders and their perspectives in order to create change [13].

The development of the MoTER-ACS intervention was informed by the social cognitive theory and used components of self-efficacy informational sources. The self-efficacy theory was adopted to reinforce behavior change, including problem solving, goal setting, action planning, e-diaries, self-monitoring, educational instructions, role modeling, and health care providers’ persuasion.

Self-efficacy stands at the core of the social cognitive theory and consists of all the thoughts that affect human functioning [17]. Self-efficacy is defined as an individual's belief in his or her capacity to achieve behaviors necessary to produce specific performance skills [17]. Self-efficacy reflects confidence in the ability to control one's own motivation, behavior, and social environment [17]. MoTER-ACS pedagogical principles were based on self-efficacy elements, including mastery experience, role modeling, and verbal persuasion. Previous research has recommended using a combination of 3 to 4 sources of the theory to promote a stronger sense of self-efficacy and a greater willingness to undergo behavioral change and thus produce optimal results [18]. Mastery experience was implemented by asking participants to self-monitor and record their health measures (for example, BP and BW) and lifestyle parameters, such as smoking, drinking alcohol, and consuming fruits and vegetables.

Role modeling through observing others’ actions is considered effective in increasing self-efficacy [19]. Peer role modeling was reinforced through educational materials linked to the Australian Heart Foundation website and the stories of people who had similar conditions. This aimed to resemble role models and encourage patients’ engagement with the intervention. A further source of self-efficacy, verbal persuasion, was applied to the intervention through motivational SMS text messages and feedback from health care professionals.

### Platform Development and Customization

The purpose of the MoTER-ACS was to overcome accessibility barriers and enable patients to either receive traditional face-to-face outpatient follow-up or a smartphone-based approach. The major platform design objectives were to provide patient-centered educational materials and symptom monitoring, support, and feedback by health care professionals based on the theory-driven strategies. To achieve these objectives and extend the platform, we conducted prestudy surveys that consisted of questionnaires among a group of patients (n=30), a focus group discussion with health care professionals (n=10), and an online survey among cardiologists (n=15). The study procedure was approved by the relevant human research ethics committee.

To address the research methodology as an iterative process and to modify the MoTER-ACS intervention, the survey among patients and the focus group with health care professionals will be repeated following feasibility testing. Repeating the prestudy surveys aims to determine any modifications required.

### Prestudy Survey

#### Patient Survey

We conducted a systematic review to investigate smartphone-based educational interventions for patient self-management. The review also explored the mHealth structures and strategies (including format, interactivity, use of theory, duration of education, and health care professionals’ follow-up) of the educational interventions, along with any documented theory or framework that informed the design of such interventions. The results of the systematic review were published separately [15].

After conducting the systematic review, we explored the perceived learning and educational needs of patients with ACS (n=30) after an episode of MI or angina, and we assessed their health-related literacy. We used validated questionnaires and recruited a convenience sample of patients who attended an outpatient clinic at a metropolitan hospital located in Brisbane, Australia. The participant characteristics are provided in Multimedia Appendix 1. Since we could only involve patients during outpatient clinic visits, it was not possible to invite a group of patients to attend a focus group discussion at a specific day and time. In addition, many of these patients travelled about an hour to come to the clinic, so we decided to conduct a survey with each patient individually. After signing the consent form, participants completed a demographic information questionnaire, the Cardiac Patients Learning Needs Inventory (CPLNI) [20,21], and the Australian version of the Short Test of Functional Health Literacy in Adults (S-TOFHLA) [22].

Statistical analysis was performed using IBM SPSS Statistics version 21 for Windows (IBM Corp). Descriptive statistics (mean, standard deviation, frequencies, and percentage) were used to describe the study sample and patients’ perceptions of learning needs and health literacy.

The CPLNI is a Likert scale consisting of 38 items. Participants were asked to score each question from 1 to 5 according to the level of importance (1=not important, 2=somewhat important, 3=moderately important, 4=important, and 5=very important). According to the CPLNI scoring method, we assessed patients’ learning needs based on the mean score of each domain, which ranged from 1 to 5. Higher scores reflected greater learning needs. To identify the educational topics most important for patients with ACS, the mean of each of the 8 educational topics was calculated and the topics were ranked from highest to lowest. Reasons for MI and signs and symptoms were ranked as the most important topics for learning, followed by medications management, lifestyle factors, diet, psychological factors, and lastly, physical activity (Multimedia Appendix 2). The identified topics were considered as part of the intervention’s educational information. Therefore, we sought authorization from the Australian Heart Foundation to embed links from their website in the MoTER-ACS intervention. Furthermore, we included related video clips based on the educational topics identified by patients.

Monitoring health literacy assists health care providers in identifying patients who have difficulties with the educational instructions of cardiac rehabilitation and require further education to obtain adequate disease-related knowledge [22]. In this study, we observed adequate health literacy among the study participants. The S-TOFHLA results were scored as inadequate (0-16 correct answers), marginal (17-22 correct answers), and adequate (23-36 correct answers) health literacy. Of 30 participants, 1 (3%) was identified as having inadequate health literacy and 28 (94%) were identified as having adequate health literacy. One participant found the survey frustrating and did not answer it. No patient was identified as having marginal health literacy.

#### Focus Group

The purpose of the focus group was to identify components of MoTER-ACS for the postdischarge management of patients. In May 2017, we conducted a focus group with health care professionals (n=10) from a metropolitan hospital located in Brisbane, Australia. Participants from a multidisciplinary team including cardiologists, nurse practitioners, clinical nurses, and a physiotherapist contributed to a 1-hour discussion by responding to 8 questions on the applicability of smartphone-based educational and health interventions (Multimedia Appendix 3). Transcriptions of the audiotaped session were generated and then imported into NVivo 11.0 (QSR International) for thematic analysis. The NVivo software was used for managing and organizing data, facilitating the process of analysis, identifying themes, collecting insight, and drawing conclusions [23]. Based on the focus group questions, relevant codes were assigned to the text fragments, reflecting the words spoken by the participants in a more abstract way. The coding process assisted with structuring and revealing themes within the text. Four major themes and their subthemes emerged from the qualitative analysis. The complete results of the focus group were published in 2018 [24].

Health care providers indicated that comprehensive education on diet, particularly by providing a daily meal plan, is essential for patients with ACS. For ACS symptoms, clinicians recommended mainly focusing on educating patients instead of monitoring chest pain and shortness of breath daily, as these are subjective and may not sufficiently inform clinicians. Results of the focus group also suggested that monitoring health measures such as BP and BW may result in increased awareness of patient physical health, yet may not be sufficient to support patients with ACS via the smartphone-based intervention [24]. Therefore, monitoring pain, emotional status, and other health measures was recommended. Real-time support via FaceTime or video conferencing was indicated to be motivational and supportive for patient engagement with the intervention. Higher age, low educational level, and lack of computer skills were identified as potential barriers for patients with ACS to engage with the smartphone-based intervention [24]. The items related to educational topics, meal plans, and measuring health parameters identified by health care professionals shaped different parts of the MoTER-ACS intervention.

#### Cardiologist Survey

We used an online survey to investigate specialists’ postdischarge practices for patients with ACS. Investigating cardiologists’ perspectives aimed to provide insight on methods of patient treatment and follow-up. This assisted us in translating specialists’ practices into knowledge for developing a smartphone-based postdischarge intervention.

We developed the online survey based on the key objectives for developing a model of care introduced by the US Department of Health and Human Services’ Center for Disease Control and Prevention [25]. The survey consisted of 12 multiple choice questions that covered topics such as systematic management to meet the needs of patients with ACS, multidisciplinary care coordination and communication, partnership between patients and cardiologists, and patient risk assessment. Through SurveyMonkey, the survey was sent to the email addresses of cardiologists from the hospital located in Brisbane, Australia. Of 45 cardiologists, 15 responded to the survey. Descriptive statistics were used to analyze the responses. The frequency of responses to each question were calculated and responses with higher frequency were considered within the intervention design if applicable.

The specialists considered regular medical assessment and follow-up at an outpatient clinic an effective method for the prevention of ACS readmission in the first 12 months. Based on the results of the online survey, the majority of cardiologists provided their services based on the patient’s risk level. Consultation services were provided to patients with high risk every 3 or 6 months. Patients with low risk were followed up every 12 months. The results also showed that follow-up appointments for patients with ACS were mainly to assess response to treatment, prevent readmission, provide support, and adjust medication (Multimedia Appendix 4).

Onset of heart failure (HF) was identified as the main reason patients with ACS presented to the hospital. Other reasons, including chest pain, shortness of breath, unstable angina, and ST-elevation myocardial infarction or non–ST-elevation myocardial infarction, were also reported by cardiologists. Based on the results of the online survey with cardiologists, monitoring symptoms related to heart failure exacerbation was considered as a tool (weekly diary) in the MoTER-ACS intervention.

## Results

### Platform Components

**Figure 1:**
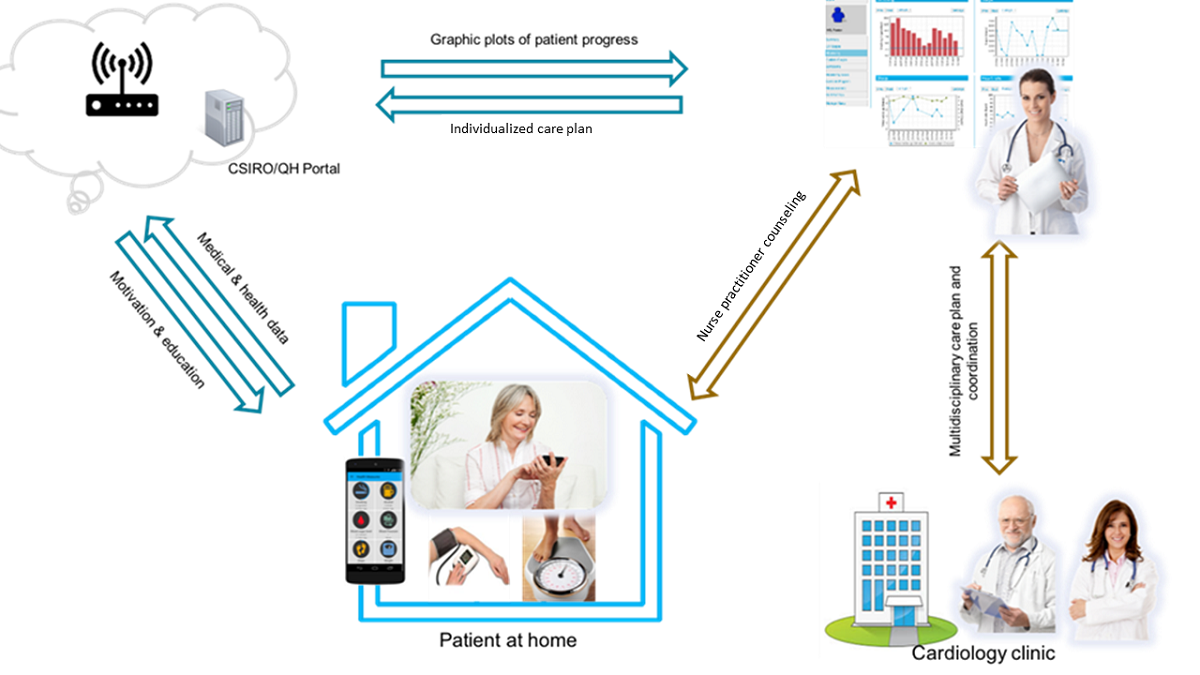
MoTER-ACS platform. CSIRO: Commonwealth Scientific and Industrial Research Organisation; MoTER-ACS: Mobile Technology–Enabled Rehabilitation for Patients With Acute Coronary Syndrome; QH: Queensland Health.

We extended the MoTER platform (Figure 1) based on the results from the prestudy. The MoTER-ACS platform consists of smartphone apps (Android and iOS), Bluetooth-enabled medical devices, and a web portal. Apps are used for providing education and personalized feedback, collecting physiological data, recording patients’ self-observations of their health-related behaviors, and facilitating health care providers’ consultations via audio or video. All the data entered into the apps by patients are synchronized daily to a web portal on a secure server with a user-friendly front-end web portal, where clinicians can regularly (every 2 to 3 days) monitor physiological data.

#### MoTER-ACS App

**Figure 2:**
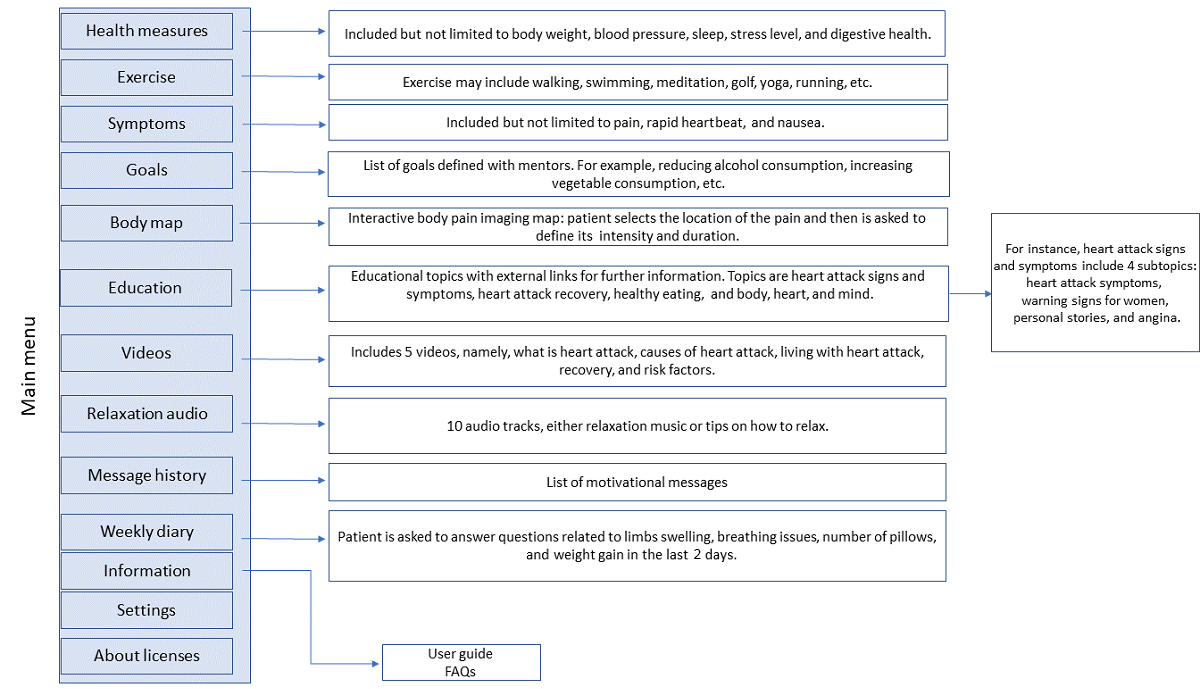
Navigation of the MoTER-ACS app. FAQ: frequently asked question; MoTER-ACS: Mobile Technology–Enabled Rehabilitation for Patients With Acute Coronary Syndrome.

The MoTER-ACS apps consist of a number of software components, including health measures, multimedia educational materials, a body pain map, a weekly diary, relaxation audio, and motivational messages. Figure 2 outlines the navigation of the MoTER-ACS app, with examples from each category.

##### Health Measures

**Figure 3:**
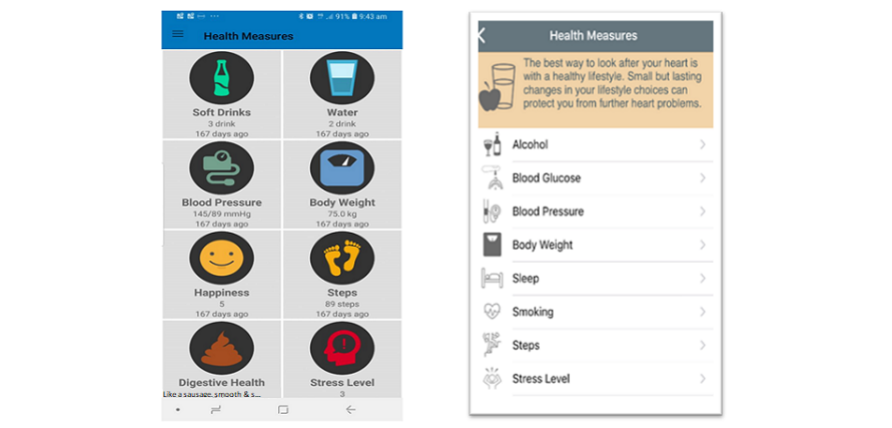
Health measures screen for iPhone.

Following enrollment, each patient sets goals (smoking, alcohol intake, etc) with the help of their nurse, or mentor, to start the program. During the intervention, the mentor discusses the patient’s progress in comparison with the set goals and assists in setting new goals. Therefore, patients report a number of health and lifestyle measures, such as BP, BW, alcohol consumption, and servings of fruit consumed (Figure 3). Patient medication adherence is measured through agreement between the patient and mentor and as part of the health measures, where patients can enter their daily medication intake. To establish agreement between the patient and mentor, the mentor asks the patient if they agree to provide information about their medications, then considers this as the patient inputs information into the MoTER-ACS app.

##### Weekly Diary

**Figure 4:**
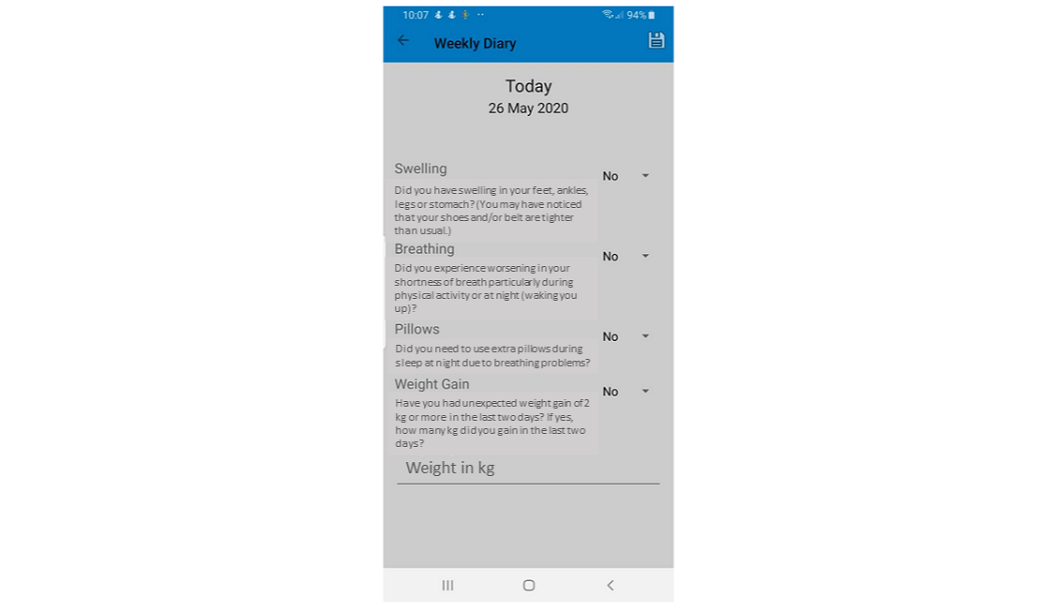
Weekly diary.

We developed a weekly diary to monitor HF symptoms. In addition to the daily monitoring of different health measures, patients are asked to answer 4 questions related to signs and symptoms of HF exacerbation, including swelling of the limbs, breathing patterns, number of pillows (when sleeping), and weight gain (more than 2 kg in the past 2 days). The symptoms are recorded weekly and the results are reported to specialists (Figure 4).

##### Educational Materials

**Figure 5:**
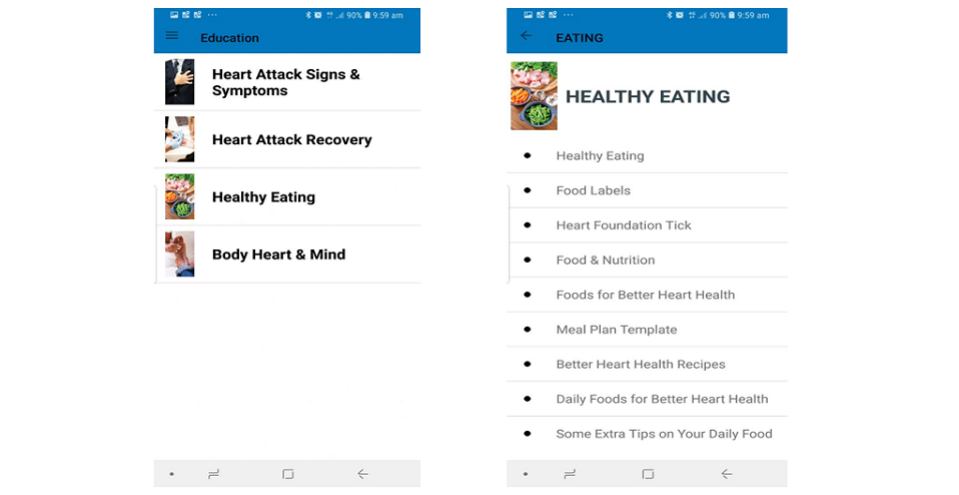
Educational materials.

In addition to 5 video clips that provide information on CVD conditions, patients have access to a wide range of information through links to the Australian Heart Foundation website, including MI signs and symptoms, MI recovery, healthy eating, and body, heart, and mind (Figure 5).

Nutritional education has a beneficial impact on the dietary habits and nutritional knowledge of patients with CVD [26]. Hence, to encourage healthy eating, we developed diet-related instructions that cover topics such as foods that are better for heart health, daily meal plans, and tips to prepare daily foods and recipes.

##### Body Pain Map

**Figure 6:**
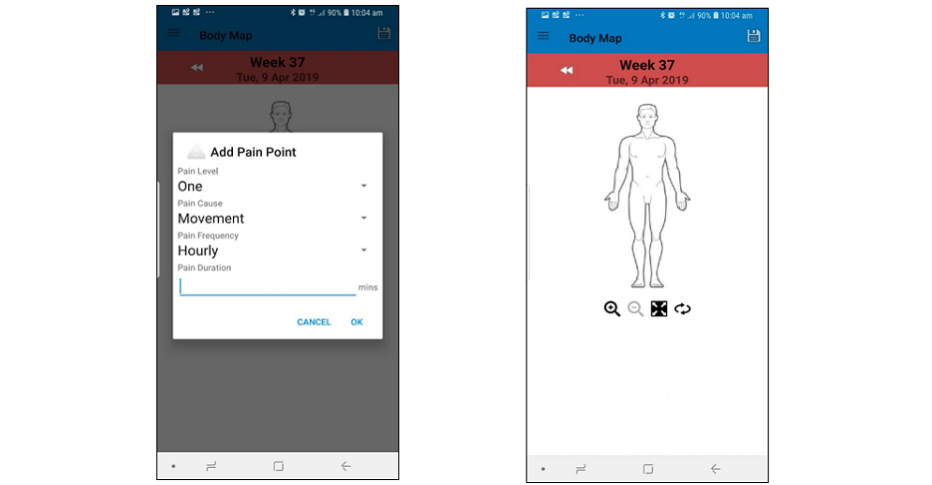
Body pain map screen on Android phone.

Pain interferes with many daily activities, and the goal of pain management is to reduce the effect of pain on patient function and quality of life, including the ability to resume daily activity, maintain a positive mood, and get adequate sleep. The body map is a tool designed for patients to identify the location of their pain and score their pain level from 0 to 10. We developed the pain assessment tool to identify pain characteristics by asking questions about pain cause, intensity, aggravation factors, frequency, and duration (Figure 6).

##### Relaxation Audio

To decrease patients’ emotional stress, the MoTER-ACS app contains relaxation audio. The audio was developed by the Australian Cancer Council, and authorization to use the audio in the MoTER-ACS intervention was obtained. The audio consists of 10 tracks, including exercises for relaxation, practical tips, and types of relaxation.

##### Motivational Messages

**Figure 7:**
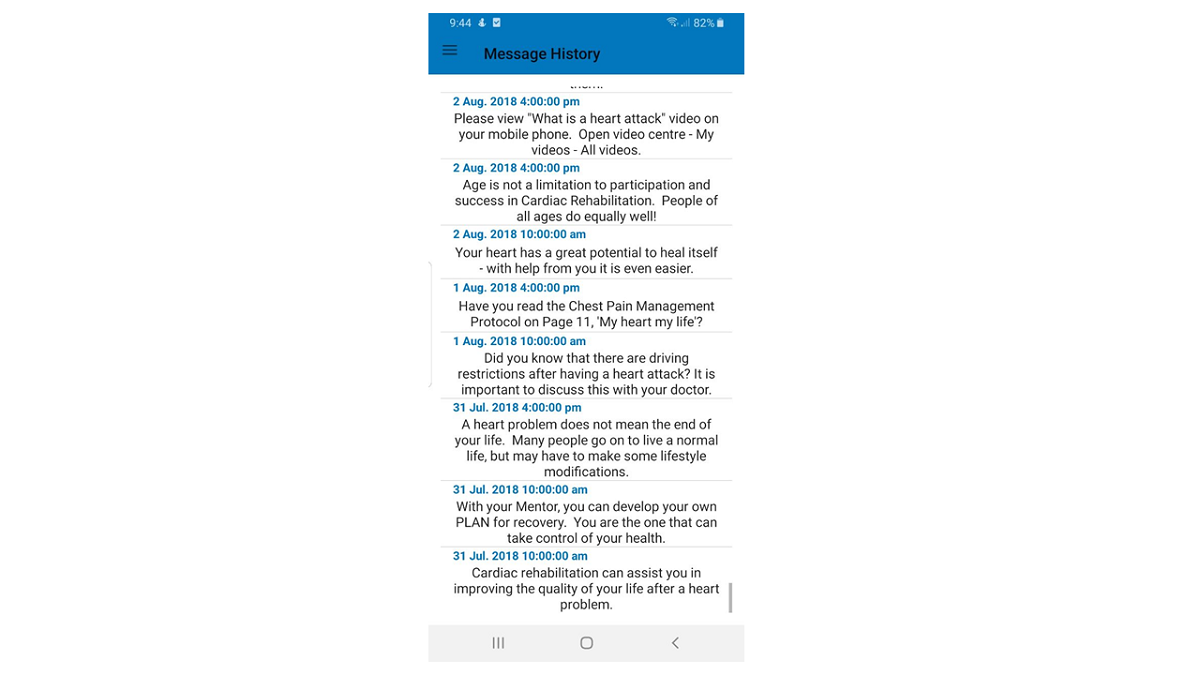
Motivational messages.

Previous research has shown that mHealth studies that reported low patient adherence used more basic and repetitive content, while successful studies used several educational and motivational strategies to engage users (ie, tailored or personalized messages) [27]. Based on health care professionals’ input, a collection of motivational messages was developed to deliver through SMS text messages throughout the program. Message content was adapted from a previous study that demonstrated positive effects on lifestyle behaviors [14]. Daily SMS text messaging aims to enhance patients’ self-management, competence, and relatedness. However, in this study, the messages were general and not tailored or personalized (Figure 7).

#### Web Portal

**Figure 8:**
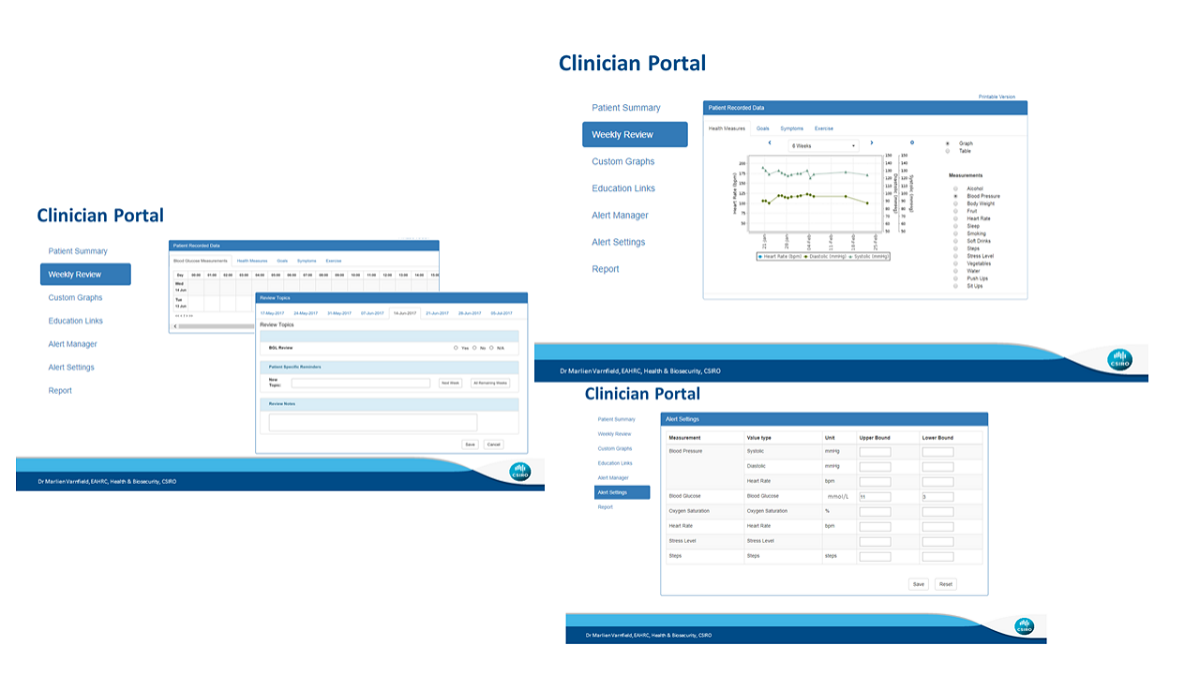
MoTER-ACS web portal screenshot. MoTER-ACS: Mobile Technology–Enabled Rehabilitation for Patients With Acute Coronary Syndrome.

The web portal enables mentors to provide patients with real-time individualized coaching, feedback, support, and instructions related to cardiovascular symptoms. Data gathered from the MoTER-ACS smartphone app are linked to the web portal, and after reviewing patients’ physiological data and health measures, mentors can provide their feedback and support through phone calls or FaceTime (Figure 8).

The MoTER-ACS platform ensures user privacy by registering a user account into a database on a secure server. The web portal server is hosted in an Amazon Web Services Virtual Private Cloud managed by the Commonwealth Scientific and Industrial Research Organisation (CSIRO). The Android and iOS apps are publicly available via Google Play and the App Store for patients to download and register. However, smartphone- and web-based app functionalities are contingent on authentication with the secure web server in order to prevent unauthorized use of the MoTER-ACS platform, and all data transmission is encrypted.

### Pilot Testing

MoTER-ACS provides an alternative model of care to a postdischarge clinic, and it is essential to determine how the intervention compares with traditional programs before it can be implemented as routine practice. Therefore, a pilot randomized controlled trial is planned to test the feasibility of the MoTER-ACS platform with a small group (N=54) of patients with ACS for 12 weeks. The trial will aim to test aspects of the platform and intervention content design, such as educational instructions, self-management interventions, and health care providers’ support. The pilot study will be conducted based on the adopted framework and assess several outcome measures, such as participants’ compliance and adherence to the intervention, modifiable cardiovascular risk factors, medication adherence, and health status. Based on the results of the pilot randomized controlled trial, patient survey, and focus group discussion (as a poststudy activity), the MoTER-ACS intervention will be modified and a subsequent larger randomized controlled trial with longer follow-up will be conducted to observe patients over a longer period.

## Discussion

### Principal Findings

This paper outlines the development and extension of an evidence- and theory-based mHealth intervention to support patients with ACS. Advanced sensor and smartphone technologies overcome common accessibility barriers that limit patient support. mHealth interventions can potentially meet the needs of patients who are unable or unwilling to attend outpatient clinics following discharge from the hospital [28]. MoTER-ACS provides educational instructions, self-management interventions, and health care providers’ support. We aimed to close the gap between hospital- and home-based postdischarge programs by proposing an alternative model of health care delivery.

The incorporation of social and behavioral theory in apps is crucial in developing complex interventions [29]. However, there is a lack of theory-based mHealth interventions, even though some theories of behavior change are validated in evidenced-based interventions for primary and secondary prevention [30,31]. To develop the MoTER-ACS intervention, the self-efficacy theory (drawn from the social cognitive theory) was adopted to reinforce behavior change, including problem solving, goal setting, action planning, e-diaries, self-monitoring, educational instructions, role modeling, and health care providers’ persuasion.

The prestudy surveys provided information on patients' perceived learning needs and health-related literacy, which was required for developing mHealth educational interventions. Prior to developing educational interventions, assessing and evaluating patients’ learning needs is essential, and it is evidenced that adopting a structured educational plan increases the likelihood of a successful recovery [32]. Furthermore, engaging patients in the development phase and identifying their needs and preferences empowers their enthusiasm for learning and therefore assists health care providers in developing patient-centered interventions.

The focus group discussion with health care professionals resulted in useful feedback regarding content and features for the development of the MoTER-ACS intervention. The educational materials and self-management interventions identified from the prestudy surveys and systematic reviews [15,28] were incorporated into the MoTER-ACS platform to engage patients with the intervention and improve their health outcomes after discharge from the hospital. The MoTER-ACS app contains multimedia educational instructions and tools to support self-management. Educational materials with diet information could influence patient knowledge and provide guidance to follow healthy diet instructions, tips, and recipes.

Previous research has demonstrated the benefits of tracking health behaviors through mHealth. Self-monitoring, goal setting, and feedback are recommended for tracking because they are likely to increase patients’ engagement in their personalized care and offer health care providers assessments of their patients’ daily activity patterns [11]. A recent systematic review and meta-analysis of remotely delivered interventions using self-monitoring and feedback demonstrated a significant effect on patient behavior change [33]. Therefore, we considered symptom monitoring, goal setting, and feedback from health care providers within the MoTER-ACS intervention.

A systematic review of mHealth pain management showed that mobile apps are beneficial for patients, particularly those in outpatient clinics, and that both patients and health care providers were satisfied with apps that provided pain management tools [34]. Furthermore, previous research has shown that a smartphone pain diary facilitates gathering more accurate and complete pain ratings [35]. Hence, a body pain map for the MoTER-ACS app was developed to identify pain and its characteristics.

Motivational messages are delivered as part of the MoTER-ACS intervention. It is evidenced that 4 SMS text messages per week are effective in improving patients’ health outcomes [36]. Additionally, personally tailored and interactive interventions seem to be more efficacious, especially when users can choose when to receive messages [36].

Although mHealth can potentially improve patients’ self-management, adoption of such technologies by adults 50 years or older is limited by age-related barriers. Higher age, physical decline, comorbidities, and low health literacy have been identified as mHealth engagement barriers [37]. Despite older adults’ interest in using mHealth, current evidence reports that their usage and adoption of such interventions are inconsistent [38]. Therefore, facilitators and barriers potentially influencing older adults’ acceptance of mHealth must be considered [37]. In addition to providing flexible tools to engage patients via written, verbal, or video interactions, there is a need to consider how individuals without advanced technical skills will interact with the app or participate in mHealth interventions [39]. Previous research has shown that most participants expect in-person training on the use of the mHealth app in addition to on-demand online help, phone support, or support from family and friends [40-42]. Therefore, in-person training on using the mHealth intervention and the involvement of family and caregivers could be solutions to improve engagement with the MoTER-ACS intervention.

### Strengths and Limitations

Well-designed educational strategies grounded in theory and contemporary evidence are crucial in the development of complex interventions [15]. We used a theoretical framework to form the important constructs of the MoTER-ACS intervention and fully integrated it into all parts of the platform. We conducted the prestudy surveys to enhance the intervention design before embarking on feasibility testing. However, the patient survey was limited by the recruitment of a small sample of 30 patients with ACS from a metropolitan hospital. This limits the generalizability of the study results compared with sampling from other settings, including regional and remote areas. A larger sample size and nonurban hospital may provide precise information about patients' learning needs and preferences; however, the results of this study are consistent with previous research that examined cardiovascular patients’ educational needs [43].

The MoTER-ACS platform provides comprehensive educational and self-management interventions based on the secondary prevention components recommended by national guidelines. The MoTER-ACS app is designed for both Android and iOS smartphones. Moreover, the flexible platform architecture enables rapid integration of new smartphones and wearable and nonwearable sensor capabilities as they become available.

Learning to use a smartphone and health-measuring devices represents a potential barrier for older adults [37]. Although we aimed to design the MoTER-ACS app to be user-friendly, a dedicated training module is required to familiarize patients with the technology. However, some patients may not be able to overcome the technological barriers. Therefore, involving family and caregivers could be a solution to overcome these barriers [44].

### Conclusion

The MoTER-ACS platform extends the capabilities of the previous MoTER platform and provides an alternative model of care for postdischarge follow-up of patients with ACS. If proven effective, this research would enable clinicians to overcome the accessibility barriers of traditional hospital-based programs by providing mHealth follow-up. Mobile technology provides an ideal platform for the delivery of health care services and could easily be applied to the prevention and management of other chronic diseases.

## Appendix

Multimedia Appendix 1 Patient Participants’ characteristics.

Multimedia Appendix 2 Patient Participants’ responses- CPLNI.

Multimedia Appendix 3 Healthcare providers’ characteristics.

Multimedia Appendix 4 Cardiologists' survey.

## Abbreviations

ACS: acute coronary syndrome
BP: blood pressure
BW: body weight
CPLNI: Cardiac Patients Learning Needs Inventory
CSIRO: Commonwealth Scientific and Industrial Research Organisation
CVD: cardiovascular disease
HF: heart failure
mHealth: mobile health
MI: myocardial infarction
MoTER: Mobile Technology–Enabled Rehabilitation
MoTER-ACS: Mobile Technology–Enabled Rehabilitation for Patients With Acute Coronary Syndrome
S-TOFHLA: Short Test of Functional Health Literacy in Adults
