# Supplementary material for: A Smartphone App for Patients With Acute Coronary Syndrome (MoTER-ACS): User-Centered Design Approach
Source: JMIR Form Res. 2020 Dec 18;4(12):e17542. doi: 10.2196/17542 (PMC7775820; doi:10.2196/17542)
Supplement: Multimedia Appendix 1 [file formative_v4i12e17542_app1.docx]

# Appendix 1

Patients’ Sample characteristics

| **characteristics** | **Value** |
| --- | --- |
| **Age in years, mean (SD)** | 62 (8) |
| **Gender, n (%)** |  |
| Male | 26 (86) |
| Female | 4 (13) |
| **Marital status, n (%)** |  |
| Widowed/divorced | 6 (20) |
| Married | 20 (66) |
| De facto/other | 4 (13) |
| **Living arrangements, n (%)** |  |
| Live alone | 6 (24) |
| Live with others | 24 (80) |
| **Highest Level of education,** **n (%)** |  |
| Less than 12 years | 12 (40) |
| High school diploma | 7 (23) |
| Some college/associate degree | 9 (30) |
| Post graduate degree | 2 (6) |
| **Overall perceived health, n (%)** |  |
| Excellent | 7 (23) |
| Good | 14 (46) |
| Fair | 9 (30) |
| Poor | 0 (0) |
| **Health Literacy, n (%)** |  |
| Adequate | 28 (93) |
| Inadequate | 1 (3) |
| **Medication treatment, n (%)** |  |
| Antiplatelet | 25 (83) |
| ACE inhibitors | 17 (56) |
| Beta blockers | 15 (50) |
| Calcium channel blockers | 4 (13) |
| Statins | 20 (66) |
| Antianginal | 25 (83) |
