# Supplementary material for: A Smartphone App for Patients With Acute Coronary Syndrome (MoTER-ACS): User-Centered Design Approach
Source: JMIR Form Res. 2020 Dec 18;4(12):e17542. doi: 10.2196/17542 (PMC7775820; doi:10.2196/17542)
Supplement: Multimedia Appendix 2 [file formative_v4i12e17542_app2.docx]

Appendix 2

Table 1 CPLNI participants’ responses

| Educational topics | Participants’ answers (mean) | Rank |
| --- | --- | --- |
| Reasons for Myocardial Infarction | 4.63 | 1 |
| Signs and symptoms of Myocardial Infarction | 4.60 | 2 |
| Medication management | 4.50 | 3 |
| Lifestyle factors | 4.46 | 4 |
| Post discharge support | 4.39 | 5 |
| General rule diet | 4.37 | 6 |
| Psychological issues after Myocardial Infarction | 4.33 | 7 |
| General Physical activity rules | 4.30 | 8 |
