# Supplementary material for: A Smartphone App for Patients With Acute Coronary Syndrome (MoTER-ACS): User-Centered Design Approach
Source: JMIR Form Res. 2020 Dec 18;4(12):e17542. doi: 10.2196/17542 (PMC7775820; doi:10.2196/17542)
Supplement: Multimedia Appendix 3 [file formative_v4i12e17542_app3.docx]

# Appendix 3

Healthcare providers’ characteristics

| **characteristics** | **Value** |
| --- | --- |
| **Age in years, mean (±SD)** | 47 (8) |
| **Gender, n (%)** |  |
| Male | 5 (50) |
| Female | 5 (50) |
| **Highest Level of education,** **n(%)** |  |
| Less than 12 years | 0(0) |
| High school diploma | 1 (10) |
| Some college/associate degree | 0 (0) |
| Post graduate degree | 9 (90) |
| **Total Household income/year,** **n** **(%)** |  |
| Less than $ 20.000 | 0 (0) |
| $ 20-40.000 | 0 (0) |
| $ 40-80.000 | 1 (10) |
| $ 80-180.000 | 9 (90) |
| Prefer not to answer | 0 (0) |
| **Profession, n (%)** |  |
| Cardiologist | 3 (30) |
| Nurse practitioner | 2 (20) |
| Research scientist | 2 (20) |
| Clinical nurse | 2 (20) |
| Physiotherapist | 1 (10) |
