# Supplementary material for: A Smartphone App for Patients With Acute Coronary Syndrome (MoTER-ACS): User-Centered Design Approach
Source: JMIR Form Res. 2020 Dec 18;4(12):e17542. doi: 10.2196/17542 (PMC7775820; doi:10.2196/17542)
Supplement: Multimedia Appendix 4 [file formative_v4i12e17542_app4.docx]

**Post discharge management of patient with Acute Coronary Syndromes; Final Report of Cardiologists’ Survey**

# **Background**

Survey research remains the most popular source of translating knowledge to practice. We used an online based survey to investigate cardiologists’ post discharge practice for patients with Acute Coronary Syndromes (ACS). Examining cardiologists’ post-discharge practice aims to provide useful information about the interventions’ characteristics and methods of patient follow-up to improve the clinics’ effectiveness and inform decisions for future innovations. Investigating current service will assist us to translate clinical evidence into knowledge for healthcare innovations and developing a mobile phone based post-discharge support program.

# **Aims**

To propose a mobile phone based model of care for post-discharge support of patients with ACS.

# **Methods**

Based on the key objectives of developing a model of care introduced by the US department of health and human services, centre for disease prevention and control, we developed a survey to investigate current post discharge practice for ACS patients. The survey consists of 12 multiple choice questions covering the following objectives;

- Systematic management to meet the needs of ACS patients
- Multidisciplinary care coordination and communication
- Partnership between patients and cardiologists.
- Patients Risk assessment

# **Results**

Using the Survey Monkey website, the survey was sent to the email addresses of cardiologists from the Prince Charles Hospital located at Brisbane, Australia. Of 45 cardiologists, 15 responded to the survey. Descriptive statics were used to analyse the responses. For each questions, the frequency of most responded answers were calculated and presented with bar charts.

Question 1. What is the most effective method to reduce Acute Coronary Syndromes re-admissions in first 12 month?

- Regular medical based consultation at outpatient clinic
- Multi-disciplinary (nursing or allied health such as dietitian) hospital based clinic
- Nursing case management at hospital clinic
- Nursing visits to patient's home


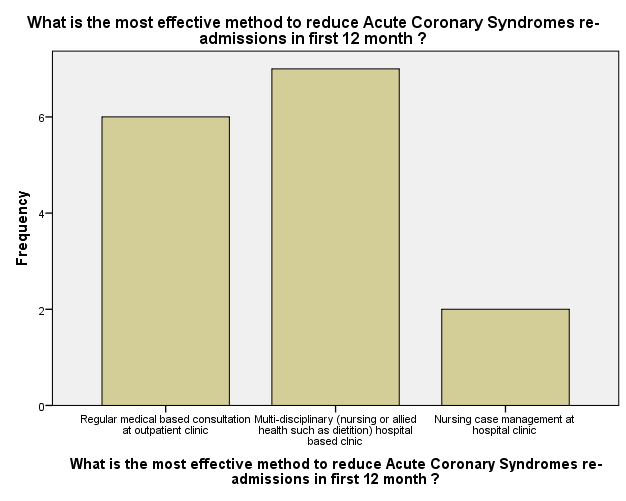


Question 2. Which of the following is the most effective intervention to reduce Acute Coronary Syndromes readmissions in the first 12 month? Please choose 3 items.

- Medication management plan
- Personal lifestyle change education
- Managing cardiovascular risk factors
- Referring patients to secondary prevention program
- Providing education for patients' self-management
- Engaging family members in patient's lifestyle change education such as smoking cessation, weight loss and increased activity


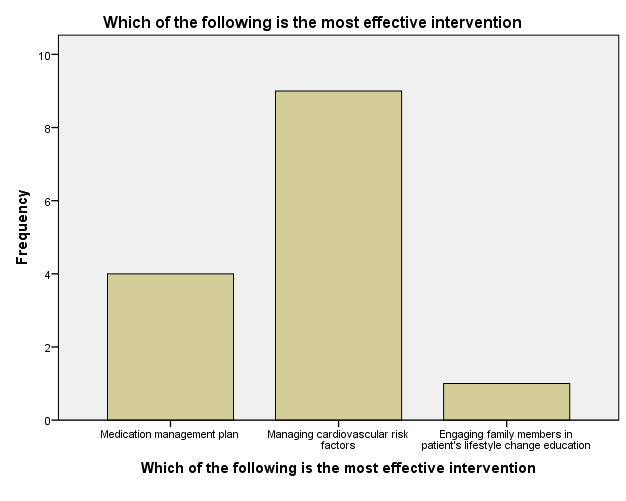


Question 3. How often do you coordinate care of your patient with other healthcare professionals?

- Always
- Often
- Sometimes
- Rarely
-
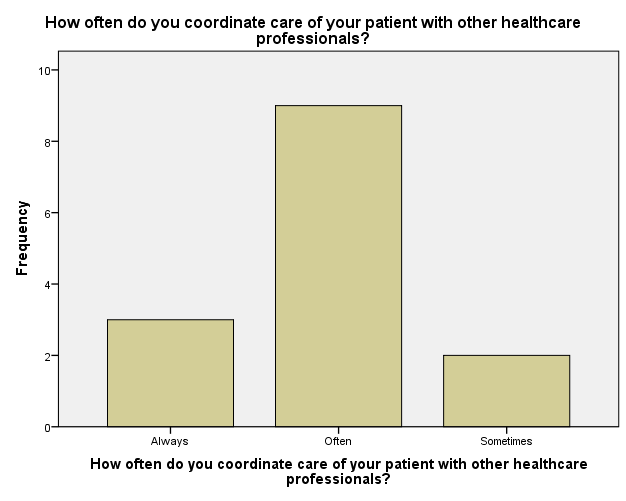
Never

Question 4. What is the best method to assist multidisciplinary (nursing, allied health, GP) team to participate efficiently in the care coordination?

- Integrated patient information via electronic health record
- Email communications
- Phone calls
- Written letters


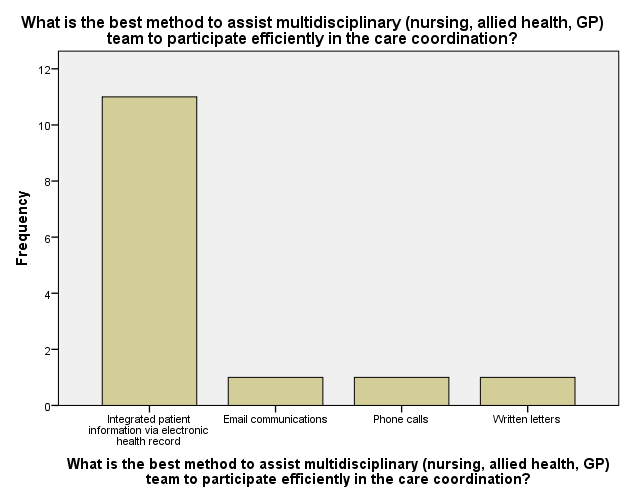


Question 5. What methods you use to coordinate care of your patients with other health care professionals? You may choose more than one answers.

- Email communications
- Phone calls
- Written letters


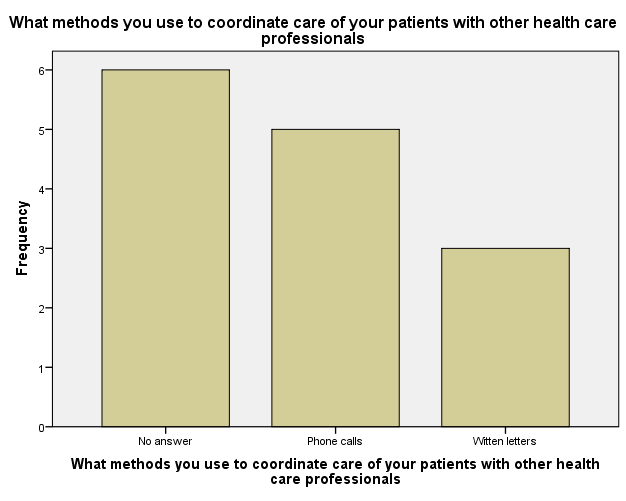


Question 6. What is the main reason that you refer a patient to other health care professionals?

- Nursing care
- Cardiac rehabilitation program
- Diet
- Exercise


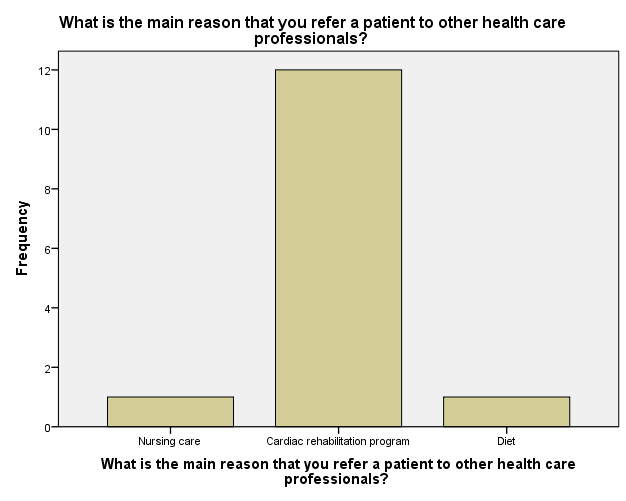


Question 7. Do you schedule consultation appointment based on patients' risk?

- Yes
- No


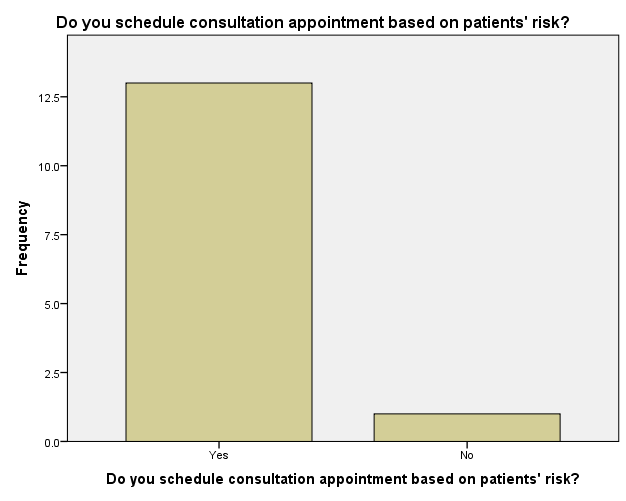


Question 8. How often do you schedule a consultation appointment for high risk patients?

- Every three month
- Every six month
- Every 12 month
- Other (please specify)


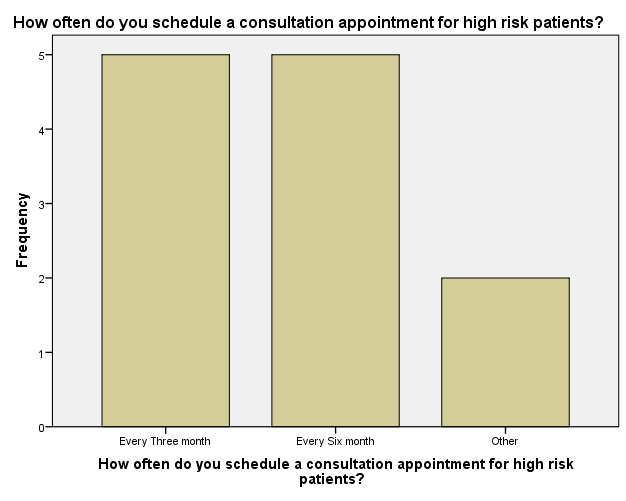


Other (please specify):

- 6 weeks then 6 monthly
- Every 3/12 initially but review overtime

Question 9. How often do you schedule a consultation appointment for low risk patients?

- Every three month
- Every six month
- Every 12 month
- Other (please specify)


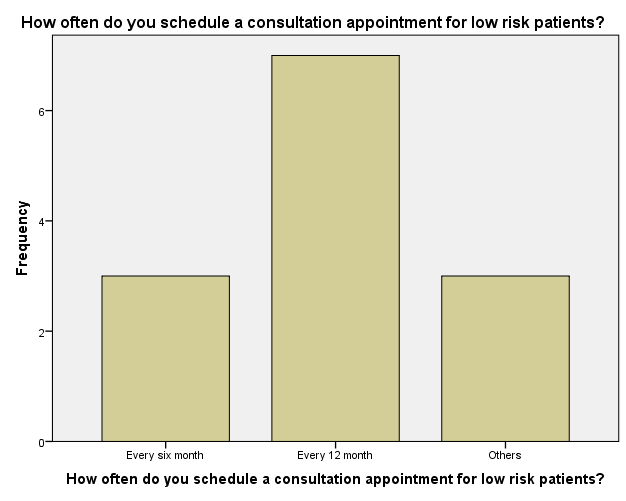


Other (Please specify):

- See GP regularly and cardiologist as-needs
- NP clinic revision
- Every 6 month until stable risk factors
- Discharge back to GP
- Every discharge to GP case

Question 10. List three most important reasons for scheduling a consultation appointment for Acute Coronary Syndromes Patients?

- To prevent re-admission
- To assess symptoms
- To assess response to treatment
- To provide support to elderly patient
- To adjust medication
- GP requested appointment
- Other (please specify)


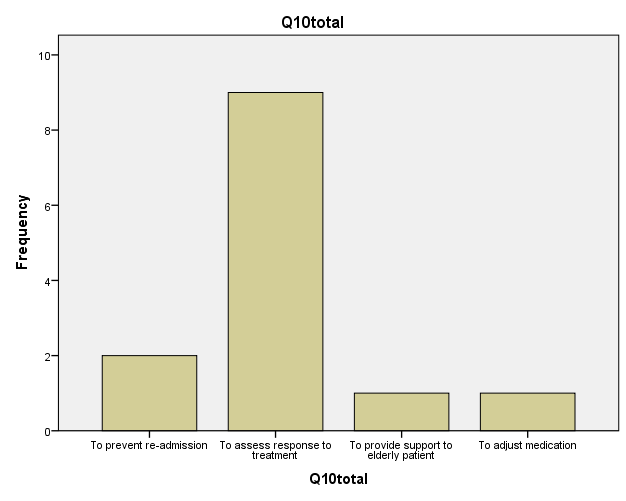


Question 11. List three most important reasons for presentation for Acute Coronary Syndromes patients?

- Chest pain
- Shortness of breath
- Onset of heart failure
- Unstable angina
- STEMI or non-STEMI
- Onset of palpitation
- Poor adherence with medication
- Poor adherence with lifestyle changes


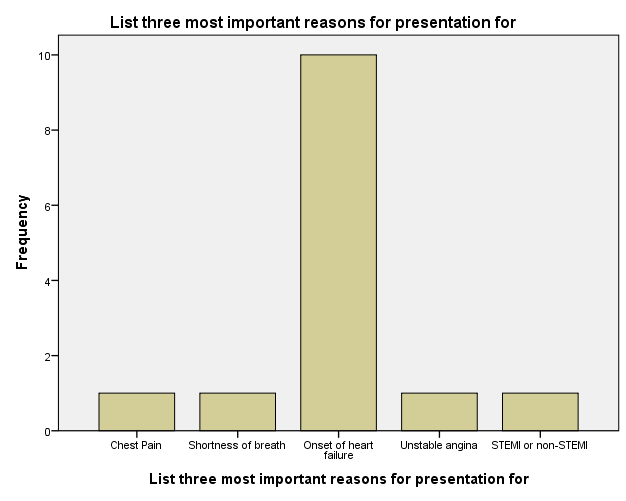


Question 12. What improvement is needed for cardiologists' outpatient clinics?

Responses:

1. high-quality EMR such as health track (not Serner)
2. Electronic records in the office.
3. medication list ready
4. better access to MDT
5. Needs to have capacity to see them early and frequently
6. more clinic space
7. improved discharge planning
8. Seamless electronic health records and access to blood tests and allied health reports in community
9. efficiency
10. more time

# Recommendations

## Multidisciplinary hospital based clinic and regular medical based follow-up

The respondents noted the importance of multidisciplinary team involvement for post-discharge management of ACS patients. They also considered regular medical based follow-up at outpatient clinic as the second effective method for prevention of ACS readmissions in first 12 month.

## Post-discharge management based on patients’ risk

Based on the results of survey, majority of cardiologists provide their services based on the patients’ risk level. Consultation services is provided to patients with high risk every three or six months. Patients with low risk are followed up every 12 months.

## Use of Integrated patient health information and medical records

The respondents frequently reported the need to capitalize on technologies and to use integrated electronic health records. Majority of cardiologists did not respond to the question which methods they use to communicate the care of their patients with other healthcare professionals.

## Referring patients to cardiac rehabilitation programs

Based on the results, the main reason that cardiologists refer ACS patients to other healthcare professionals is for cardiac rehabilitation support.

## Reason for scheduling appointment and patients’ follow-up

The respondents reported main reasons for scheduling appointments for the ACS patients were to assess response to treatment, to prevent readmission, to provide support to elderly, and to adjust medication respectively.

## Reason for ACS patients presenting at hospital

Onset of heart failure was identified as the main reason for presentation of ACS patients. Other reasons including chest pain, shortness of breath, unstable angina and STEMI or non-STEMI were also reported by cardiologists.

# Conclusion

The survey provided insight into the current practice for post-discharge management of ACS patients. The results will be used to propose a mobile phone based model of care for post-discharge management of ACS patients.
